# Simultaneous and Proportional Myoelectric Control of Multiple Degrees of Freedom in Individuals With Chronic Hemiparesis

**DOI:** 10.1109/TNSRE.2025.3599062

**Published:** 2025

**Authors:** Caleb J. Thomson, W. Caden Hamrick, Jakob W. Travis, Michael D. Adkins, Patrick P. Maitre, Steven R. Edgely, Jacob A. George

**Affiliations:** Department of Biomedical Engineering, University of Utah, Salt Lake City, UT 84132 USA; Department of Electrical and Computer Engineering, University of Utah, Salt Lake City, UT 84132 USA; Department of Electrical and Computer Engineering, University of Utah, Salt Lake City, UT 84132 USA; Department of Electrical and Computer Engineering, University of Utah, Salt Lake City, UT 84132 USA; Department of Physical Medicine and Rehabilitation, University of Utah, Salt Lake City, UT 84132 USA; Department of Physical Medicine and Rehabilitation, University of Utah, Salt Lake City, UT 84132 USA; Department of Electrical and Computer Engineering, the Department of Physical Medicine and Rehabilitation, the Department of Biomedical Engineering, and the Department of Mechanical Engineering, University of Utah, Salt Lake City, UT 84132 USA

**Keywords:** Hemiparesis, powered orthosis, human–machine interface, electromyography (EMG), motor control

## Abstract

Stroke is a leading cause of disability worldwide, with most survivors experiencing chronic motor deficits. Myoelectric orthoses, controlled by residual muscle activity from the paretic limb, can restore upper-limb function to patients. However, existing commercial myoelectric orthoses are limited to only a single hand motion with fixed force output. In the adjacent field of myoelectric prostheses, regression algorithms have enabled simultaneous and proportional position control over multiple degrees of freedom (DOFs), which in turn has improved user dexterity. Here, we explore, for the first time, the ability to regress the kinematic position of multiple DOFs in parallel from paretic muscle activity using a Kalman filter. We collected data from seven hemiparetic patients and systematically explored the root mean squared error RMSE) of kinematic predictions for various degrees of freedom. We show that proportional position control is possible for multiple hand and wrist motions and that unidirectional DOFs perform better than bidirectional DOFs. Using previously reported RMSEs from healthy participants as a benchmark, we found that 86% of hemiparetic patients achieved functional 2-DOF control, 57% achieved functional 3-DOF control, and 29% achieved functional 4-DOF control. Performance was similar across patient characteristics and different combinations of DOFs. This work demonstrates that multi-DOF regression is readily achievable for some hemiparetic patients. Restoring wrist motion, in addition to grasping, could have a substantial impact on the dexterity and independence of hemiparetic patients. As such, this work serves as an important first step towards multi-DOF assistive upper-limb exoskeletons.

## I. Introduction

Stroke is a leading cause of disability in the United States [1], with more than 795,000 individuals suffering a stroke each year [2]. Up to 80 percent of stroke-related motor deficits are in the form of upper-limb hemiparesis [3]. Another cause of hemiparesis is traumatic brain injury (TBI) [4]. Between 13 and 66% of TBIs can lead to movement disorders, including hemiparesis [5]. Hemiparesis is presented as a one-sided weakness or paralysis and is caused by damage to the central nervous system. While the deficits due to hemiparesis can vary in severity, all levels of impairment have a negative effect on quality of life and autonomy [6].

Exoskeletons, or powered orthoses, are used in both robot-assisted rehabilitation and as wearable assistive devices. Exoskeletons used in robot-assisted rehabilitation are used in short therapy sessions to rehabilitate users. Exoskeletons used as wearable assistive devices are donned daily and used to support the individual in completing activities of daily living. In both cases, the exoskeleton can be controlled using muscle signals collected through electromyography (EMG), which allows users to intuitively control the myoelectric exoskeleton using their voluntary muscle activity. Prior work has used EMG from the contralateral unimpaired limb to provide exoskeleton-driven movement of the impaired limb during bilaterally mirrored movements for rehabilitation [7], [8], [9]. However, using EMG from the impaired limb directly opens opportunities for exoskeletons to serve as a wearable assistive device in activities of daily living [10], [11], [12].

EMG can be recorded from the surface of the hemiparetic limb [13], persists in the chronic phase of hemiparesis, and can be improved over time [14], [15]. Paretic EMG has been shown to provide an intuitive control signal for powered orthoses [10], [16]. However, the ability to modulate muscle activity is diminished in chronic stroke patients [17], which can lead to abnormal muscle activations and task difficulty [18]. An additional challenge for using paretic EMG for control is that the presence of involuntary EMG increases when the individual moves another part of their arm [19]; this has been shown to decrease the accuracy of EMG-based control algorithms [20].

Due to the complexities of EMG and the abnormalities of paretic EMG [17], [19], [21], [22], current EMG control algorithms often employ a binary, “all-or-nothing” approach to move a single degree of freedom when the muscle activity exceeds a predetermined threshold. When this binary control is used to control the position of a hand exoskeleton, individuals are limited to maximally grasping or opening their paretic hand. Because there is a fixed force output from the exoskeleton, binary control makes it difficult, if not impossible, to perform fine motor actions. Variable force output is critical in tasks like manipulating fragile objects [23], preventing slips [24], and grasping under uncertain conditions [25]. Additionally, because surface EMG is a spatiotemporal summation of the underlying muscles, EMG signals are not specific enough to control multiple degrees of freedom in the hand and wrist when using binary thresholds.

In addition to binary control, other control techniques can be used to increase dexterity. These techniques include neuromuscular modeling, pattern recognition, and proportional control approaches [26].

Neuromuscular modeling maps EMG signals to muscle forces and motion predictions using physics and physiological modeling [27]. Neuromuscular modeling has been used for predicting movements in healthy individuals [28], [29], amputee populations [30], and individuals with hemiparesis [31], [32]. Neuromuscular modeling has been investigated to control exoskeletons to assist in movements of the lower limb [32], shoulder [28], elbow [33], wrist, and hand [34], [35]. Neuromuscular modeling is beneficial because it can be patient-specific [36] and has the potential to be adapted over time as patients improve [27]. However, models often require precise placement of the EMG electrodes [37] and the models can be very complex as they start to approach the complexity of the human hand [27]. Although simplified models with limited EMG inputs have been demonstrated [28], [37], real-time control for assistive exoskeletons for impaired individuals has yet to be widespread [27].

Classification approaches have been used to control multiple movements from EMG activity for research applications with exoskeletons [7], [38], [39] and commercial applications with prostheses [40], [41], [42]. Indeed, recent work has shown near-perfect classification of the attempted hand gestures of stroke patients using high-density EMG [38], [43]. However, pattern recognition systems are fundamentally limited by a single discrete class prediction. As such, they do not provide the proportional control necessary for fine force regulation. Additionally, pattern recognition requires users to classify sequential actions one after another; in the adjacent field of upper-limb prosthetics, simultaneous and proportional control of multiple degrees of freedom has been shown to outperform sequential classification in real-time tasks [44].

Proportional control of upper-limb exoskeletons has primarily been used to provide continuous estimates of force [45], [46], torque [47], [48], or velocity [12], [49]. In the adjacent field of upper-limb prosthetic control, proportional position control is common [44], [50], [51], [52], [53] and has been shown to increase performance relative to velocity control for a prosthetic hand [54]. Proportional position control is also more closely aligned with the natural encoding for hand control, which is in terms of joint position [55], [56], [57].

The few studies that have focused on proportional position control of an exoskeleton have mainly focused on the arm from the wrist up to the shoulder [49], [58]. The smaller subset of studies that do look at the hand have instead mainly explored controlling force [46], torque [48], or velocity [12]. Position control is less explored [59], [60], and has not been done extensively with stroke patients or with hand and wrist movement combinations. Grasping in combination with wrist flexion/extension and wrist pronation/supination is critical for activities of daily living [61] and is a top priority for individuals with limb impairment [62].

In our prior work, we demonstrated that high-density EMG in conjunction with a Kalman filter can provide unidirectional proportional positional control of one degree of freedom for either hand grasping or hand opening [59]. Building on this work, here we explore the scalability of this approach to simultaneous and proportional control of multiple bidirectional degrees of freedom in an offline, or human out-of-the-loop analysis. We investigated various combinations of hand and wrist movements to determine which subsets hold the most potential for real-time, simultaneous, and proportional control of a multiarticulate exoskeleton. We first show that robust unidirectional proportional position control is possible with various hand and wrist motions when performed in isolation. We also show that unidirectional proportional position control is better than bidirectional. Finally, we demonstrate that simultaneous and proportional position control is possible for up to four unidirectional degrees of freedom. As expected, performance tends to decrease as the number of controllable degrees of freedom increases, but two of the hemiparetic stroke participants achieved 4-DOF control comparable to the state of the art in prosthetics [53], [63]. Thus, this work constitutes an important step towards bringing recent advancements in simultaneous and proportional control from the field of prosthetics into the field of orthotics, which in turn could improve the quality of life for the millions of individuals with chronic hemiparesis.

## II. Methods

### A. Participant Information

Five stroke survivors and two survivors of TBI with hemiparesis were recruited for this study. Inclusion and exclusion criteria were modeled after the MyoPro (Myomo, Burlington, MA, USA) inclusion and exclusion criteria [64] as we wanted to target individuals who would potentially qualify for such an assistive exoskeleton. Briefly, individuals need to have detectable muscle activity in their forearm and spasticity measuring less than 2 in their fingers and wrist on the Modified Ashworth Scale (MAS). Informed consent and experimental protocols were carried out in accordance with the University of Utah Institutional Review Board (No. 00098851) and the Declaration of Helsinki guidelines. The average age of the participants was 45.7 ± 11.5 years. Five participants had a MAS score of 2 in their fingers; the other two had a MAS score of 1. Four of the stroke survivors suffered ischemic strokes, and one suffered a hemorrhagic stroke. Participants had an average time of 4.5 ± 3.3 years since their injury. Table I lists the demographics of the participants, including age, sex, Modified Ashworth Scale score, type of injury, and years post-injury.

### B. Electromyography Signal Acquisition

**Fig. 1.**
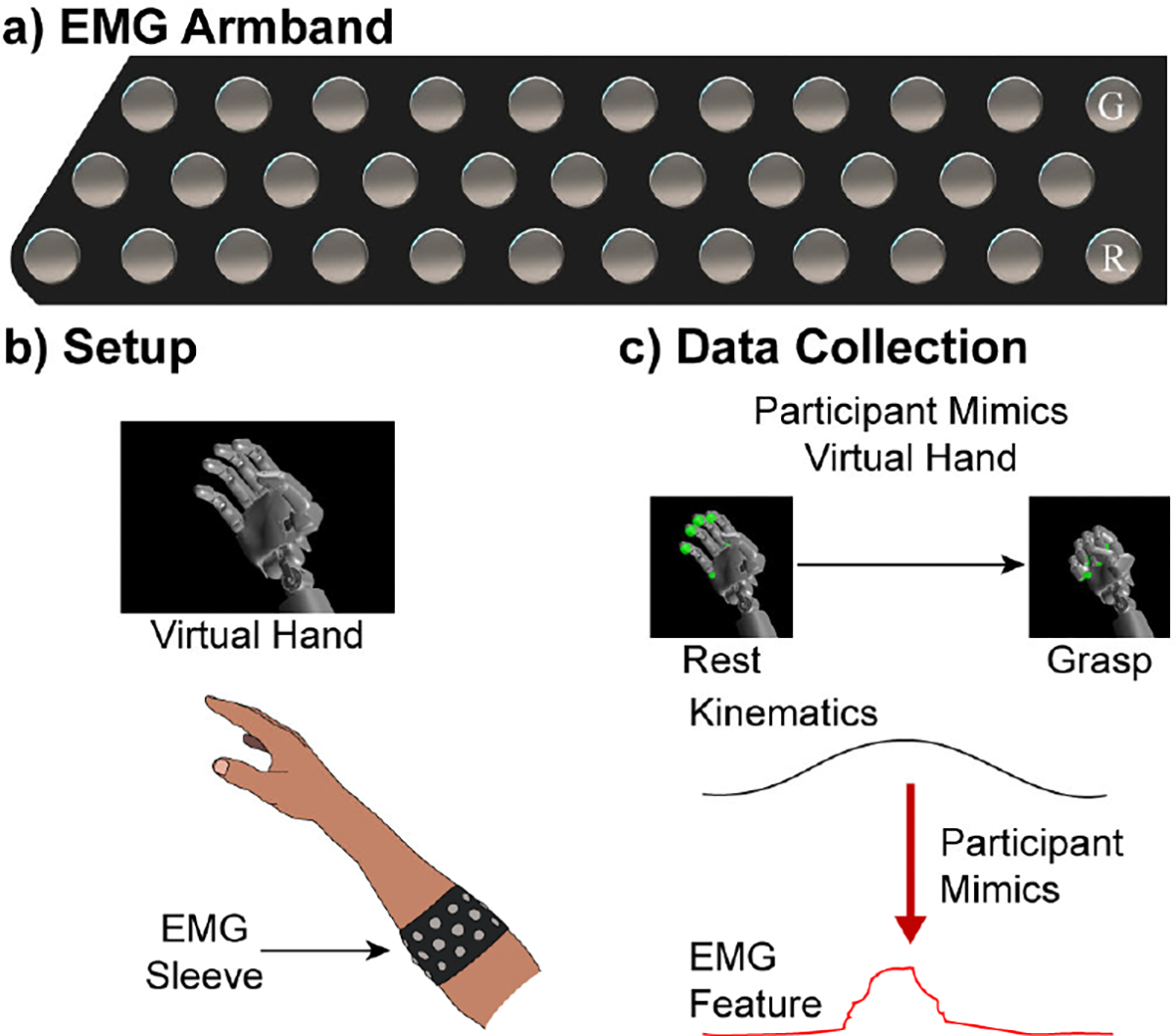
EMG Armband and data collection. a) Layout of the armband The reference and ground electrodes are marked by G and R in the illustration and would be worn near the ulna bone. b) Illustration of where the armband would be worn by the participants. It was placed over the muscle bellies in the forearm. c) The participants attempted to mimic preprogrammed kinematics of the virtual hand displayed on a computer screen and EMG was recorded in synchrony during data collection to be used in both training and testing of the modified Kalman filter.

Surface electromyography (EMG) was collected using custom EMG armbands similar to [43] and [65] (Fig. 1a). The armband was embedded with 34 nickel-plated brass electrodes soldered to a Samtec connector (Samtec, New Albany, Indiana, USA); two electrodes served as ground and reference near the ulna bone. The remaining 32 electrodes produced a single-ended channel of EMG, each using the permanent ground and reference. EMG was sampled at 1 kHz, bandpass filtered (15 Hz to 375 Hz), and notch-filtered at 60, 120, and 180 Hz using the Summit Neural Interface Processor (Ripple Neuro, LLC, Salt Lake City, UT, USA). The mean absolute value (MAV) was calculated for the 32 EMG channels and all differential pairs, resulting in a total of 528 EMG channels that were smoothed using an overlapping 300-ms window [53].

### C. Data Collection

**Fig. 2.**
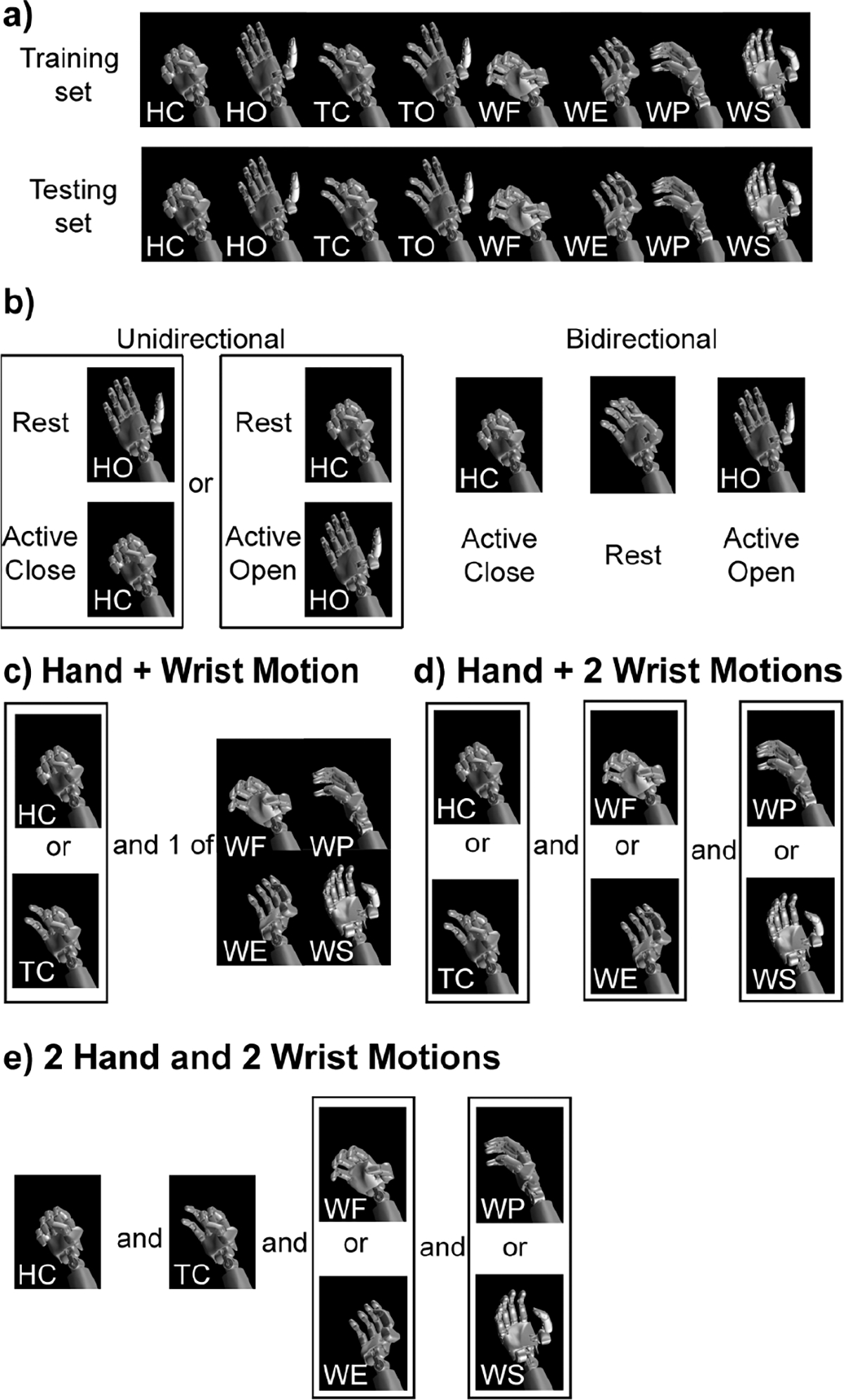
Data collection and analysis **a)** Each movement was completed during both the training dataset and the testing dataset. Movements included: full hand grasp (flexion of digits 1–5; HC), full hand open (extension of digits 1–5; HO), tripod close (flexion of digits 1–3; TC), tripod open (extension of digits 1–3; TO), wrist flexion (WF), wrist extension (WE), pronation (WP), and supination (WS) **b)** One DOF analysis looked at both unidirectional movements, e.g. just HC or HO, and bidirectional movements, e.g., HC and HO. **c)** Two DOF analysis looked at a grasping motion (HC or TC) combined with one wrist motion (WF, WE, WP, or WS). **d)** Three DOF analysis looked at a grasping motion (HC or TC), a wrist pitch motion (WF or WE), and a wrist roll motion (WP or WS). **e)** Four DOF analysis looked at two grasping motions (HC and TC), a wrist pitch motion (WF or WE), and a wrist roll motion (WP or WS).

The participants were instructed to mimic the programmed movements of a virtual bionic hand as best as they could with their paretic arm (MSMS; Johns Hopkins Applied Physics Lab, Baltimore, MD, USA) to correlate EMG activity to intended movements (Fig. 1b–c). As the participants attempted to mimic the virtual hand with their paretic hand, we recorded, in synchrony, the kinematics (joint positions) of the virtual hand and the EMG activity. The hand kinematics were computer-generated based on the rise and hold time of each movement and updated at 30 Hz with the EMG MAV signal. As in [66], we aligned the kinematic positions with the EMG signal by shifting the kinematic positions by a lag determined by cross-correlation to account for the participant’s reaction time. The participants completed two data collections, one of which would later be used for algorithm training and one of which would later be used for algorithm testing. The two datasets were identical in the movements that were attempted and included the following movements: full hand grasp (flexion of digits 1–5; HC), full hand open (extension of digits 1–5; HO), tripod close (flexion of digits 1–3; TC), tripod open (extension of digits 1–3; TO), wrist flexion (WF), wrist extension (WE), pronation (WP), and supination (WS) (Fig. 2a). Each movement was 4.4 s in duration, consisting of 0.7 s of flexion/extension away from the resting hand position, a 3-s hold-time at the maximum distance away from the resting position, and a 0.7-s relaxation returning to the resting hand position as described in [53] and [59]. Each movement was completed 10 times in each session. The participants were given 3 s of rest between movements. Each session lasted about 10 minutes. After the first session, the participants were allowed ample time to rest before collecting the second dataset to reduce fatigue.

### D. Modified Kalman Filter

We applied a Kalman Filter (KF) defined in prior work with upper-limb amputees [53], [66], [67], [68] and with stroke participants [59] to estimate and predict motor intent from the continuous EMG signals. Post-hoc modifications to this KF have been described in prior work [53], leading to the modified KF (MKF) used in this study. As in prior work [59], [66], [68], we used a threshold value of 0.2, such that the modified output would remain at zero until the absolute value of the nonmodified output was greater than 0.2. The baseline EMG MAV was subtracted from the EMG features before training and testing the KF. The EMG feature set of 528 channels was reduced to 48 channels using a stepwise Gram-Schmidt channel-selection algorithm [69], [70]. A single MKF was used to predict the position of the virtual hand. We limited outputs of the MKF between −1 and +1, where −1 corresponded to maximum extension, +1 corresponded to maximum flexion, and 0 corresponded to when the hand was at rest [52]. We used 100% of the data from the first dataset to train each MKF.

### E. Experimental Protocol

We completed this work offline, meaning users were not actively providing control. We investigated what movements and combinations yielded the best control and how many movements the MKF could regress. The datasets collected from each participant were used in this protocol. The datasets were split by movement, and different movement combinations were combined from the first dataset to train the MKF, and the same movement combinations were combined from the second dataset to test the MKF. A separate MKF was trained for each condition using the selected subset of movements from the first training dataset and tested on the same subset of movements in the second training dataset.

We first looked at each of the eight movements in isolation to verify that they could be controlled in isolation against rest. The control afforded by this unidirectional control would be akin to a voluntary-open or voluntary-close prosthesis [71]. For example, if only the hand close (HC) motion is used, the exoskeleton would keep the hand in a maximally open position by default, and the user would have to voluntarily close the hand using their muscle activity. Since we are exploring proportional position control, the hand would close to a position in proportion to the muscle activity.

Next, we examined the bidirectional movement of the four degrees of freedom: 1) hand open/close, 2) tripod open/close, 3) wrist flex/extend, and 4) wrist pronate/supinate. The control afforded by this bidirectional approach would keep the hand or wrist in a visually appealing neutral resting position. For example, if using the hand open (HO) and hand close (HC) motions bidirectionally, the hand would remain in a neural semi-closed resting position by default and then open in proportion to the muscle activity associated with hand opening and close in proportion to the muscle activity associated with hand closing (Fig. 2b).

Performance was generally worse with bidirectional movements, so we next examined the ability to control combinations of two, three, and four unidirectional movements. We limited the combinations explored to the combinations most functionally relevant (i.e., either a hand or tripod close in combination with one or more wrist motions) (Fig. 2c–2e).

### F. Data Analysis

Performance was measured as the root mean square error (RMSE) of both intended and unintended movements, as in [53], [63], and [72]. Intended movement RMSE captures the ability of the user to precisely control a given degree of freedom (DOF). It is calculated as the error between the target position and the predicted kinematic position for the DOF in which the target is nonzero (1). Where H is the number of samples during each movement attempt, $M_{m}$ DOFs were instructed to move, $x_{j,k}^{M}$ is the desired position of the *j*th DOF at the *k*th time bin, and ${\overset{ˆ}{x}}_{j,k}^{M}$ is the predicted position.

(1) $$RMSE=\sqrt{\frac{1}{HM_{m}}\sum_{k=1}^{H}\sum_{j=1}^{M_{m}}{\left(x_{j,k}^{M}-{\overset{ˆ}{x}}_{j,k}^{M}\right)}^{2}}$$

Unintended movement RMSE captures the ability of an individual to move a given DOF in isolation. It is calculated as the error between the target resting positions and the predicted kinematics for the remaining DOFs where the target is zero (2). Where $M_{s}$ DOFs were stationary and ${\overset{ˆ}{x}}_{j,k}^{S}$ was the decoded position of the *j*th stationary DOF in the *k*th time bin.

(2) $$xRMSE=\sqrt{\frac{1}{HM_{s}}\sum_{k=1}^{H}\sum_{j=1}^{M_{s}}{\left({\overset{ˆ}{x}}_{j,k}^{S}\right)}^{2}}$$

For example, consider a 2-DOF setup involving hand close (HC) and wrist pronation (WP). If the target trajectory were to perform HC in isolation, then the intended movement RMSE would be measured as the difference between the target trajectory of the five fingers and the predicted kinematic trajectory of the five fingers; unintended movement would be measured for WP, as the difference between the actual kinematic trajectory of the wrist rotation and the target resting position of the wrist rotation. For setups with more than 2 DOFs, multiple values of unintended movement are produced for each DOF; these values are averaged across DOFs to create a single value of unintended movement RMSE per trial. Unintended movement RMSE is excluded when only 1 DOF is used, as there is inherently no unintended movement when controlling a single DOF.

In subsequent analyses, we selected the best combination of DOFs per participant for the 2-DOF, 3-DOF, and 4-DOF conditions. The best combination of DOFs was determined by the combination that yielded the lowest intended and unintended RMSE. We then compared the intended and unintended RMSE against previously reported values for healthy participants (intended RMSE: 0.45, unintended RMSE: 0.04) [72]. If both the intended and unintended movement RMSE were lower than these previously reported values, the control was categorized as “functional.”

### G. Statistical Analysis

All statistical analyses were completed using the Statistics and Machine Learning Toolbox in MATLAB 2023b (MathWorks, Natick, MA, USA). Data were determined to be nonparametric through the Anderson-Darling test (*p*’s < 0.05), so nonparametric statistical analyses were performed. Separate one-way Kruskal-Wallis tests were performed to compare different movements, participants, and combinations of movements. No significance was found, so no subsequent pairwise comparisons were completed. The grouped data for one DOF unidirectional and one DOF bidirectional were also nonparametric, so a paired nonparametric *t*-test (Wilcoxon signed-rank test) was used to compare the two groups.

## III. Results

### A. One DOF Control Analysis

**Fig. 3.**
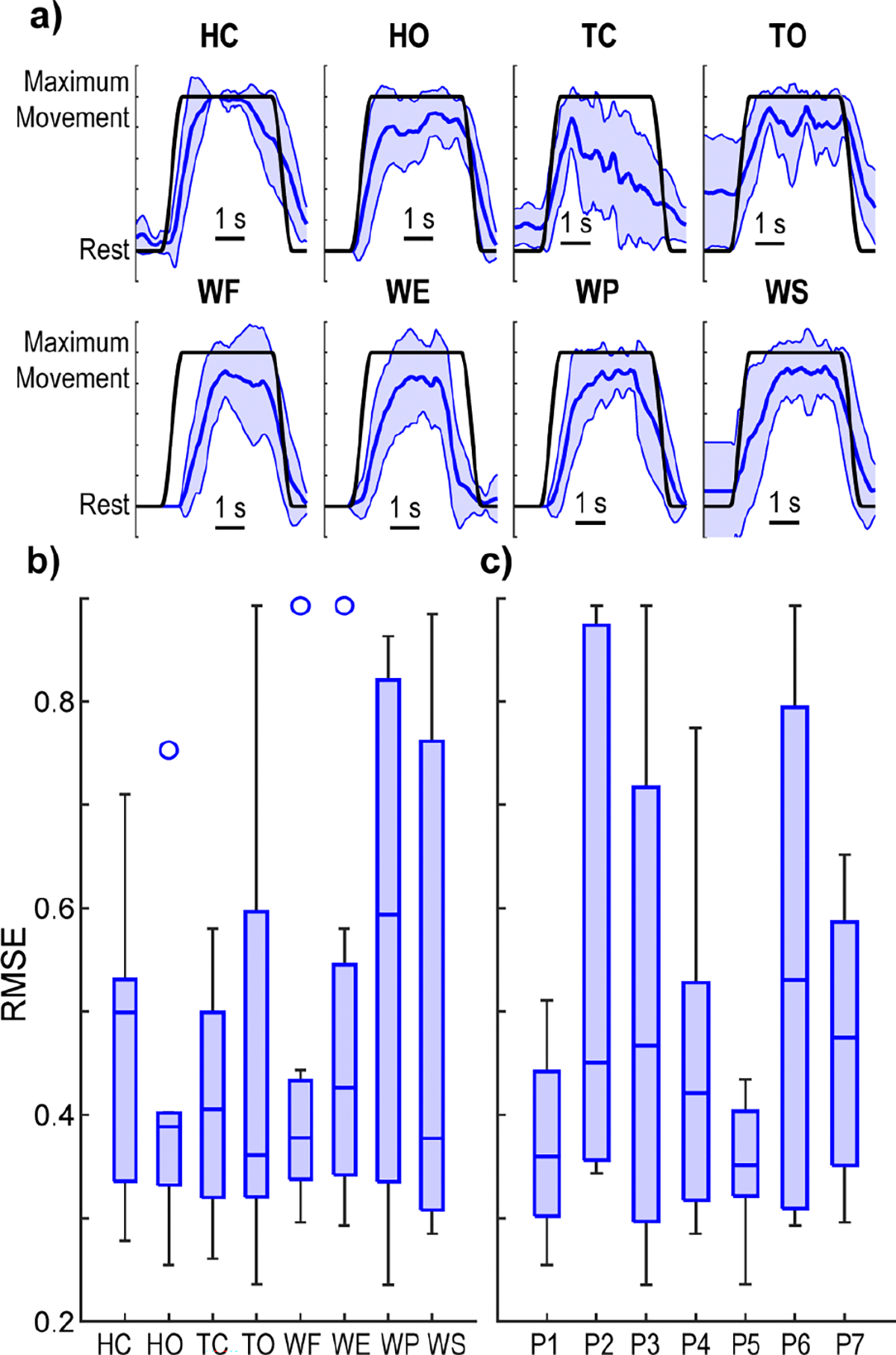
Unidirectional control of isolated hand and wrist motions. **a)** Kinematic predictions from one representative participant for each of the movements. Blue lines show the mean and standard deviation of the predicted kinematics across 10 trials of each movement from the testing dataset. Black lines show the ground-truth intended movement. The intended movement RMSEs for the example traces shown are as follows: HC = 0.28, HO = 0.25, TC = 0.51, TO = 0.34, WF = 0.44, WE = 0.44, WP = 0.33, WS = 0.38. Supplemental video S2 shows the mean traces for each movement. **b)** Boxplots of the median intended movement RMSE across participants for each movement. No significant difference was found between the movements (*p* = 0.98, Kruskal-Wallis). Data consist of one point per hemiparetic participant (N = 7). The overall median RMSE across all DOFs and participants is 0.40. **c)** Boxplots of the average intended movement RMSE across movements for each participant. No significant difference was found between the participants (*p* = 0.45, Kruskal-Wallis). Data consist of one point per movement (N = 8). Boxplots show the median, interquartile range, and most extreme non-outlier values. Circles denote outliers.

We first investigated the ability to regress the continuous kinematic position of isolated unidirectional hand and wrist motions from paretic EMG. We found that all participants were able to achieve proportional position control for each of the eight hand and wrist motions (Fig. 3a). Performance across each motion was similar, with no significant difference among the RMSEs (Fig. 3b; *p* = 0.98, Kruskal-Wallis). Supplementary Table SI has the *p*-values for the Kruskal-Wallis tests performed. Performance across participants was also similar, with no significant difference among participants (Fig. 3c; *p* = 0.45, Kruskal-Wallis). Median RMSE values were generally around 0.3 to 0.4, which is consistent with what has been reported previously with this algorithm in amputee [53] and healthy populations [72]. For comparison, an intended movement RMSE s of 0.893 would be equivalent to predicting rest for the duration of the movement (i.e., the participant does nothing).

### B. Unidirectional Vs. Bidirectional Control Analysis

**Fig. 4.**
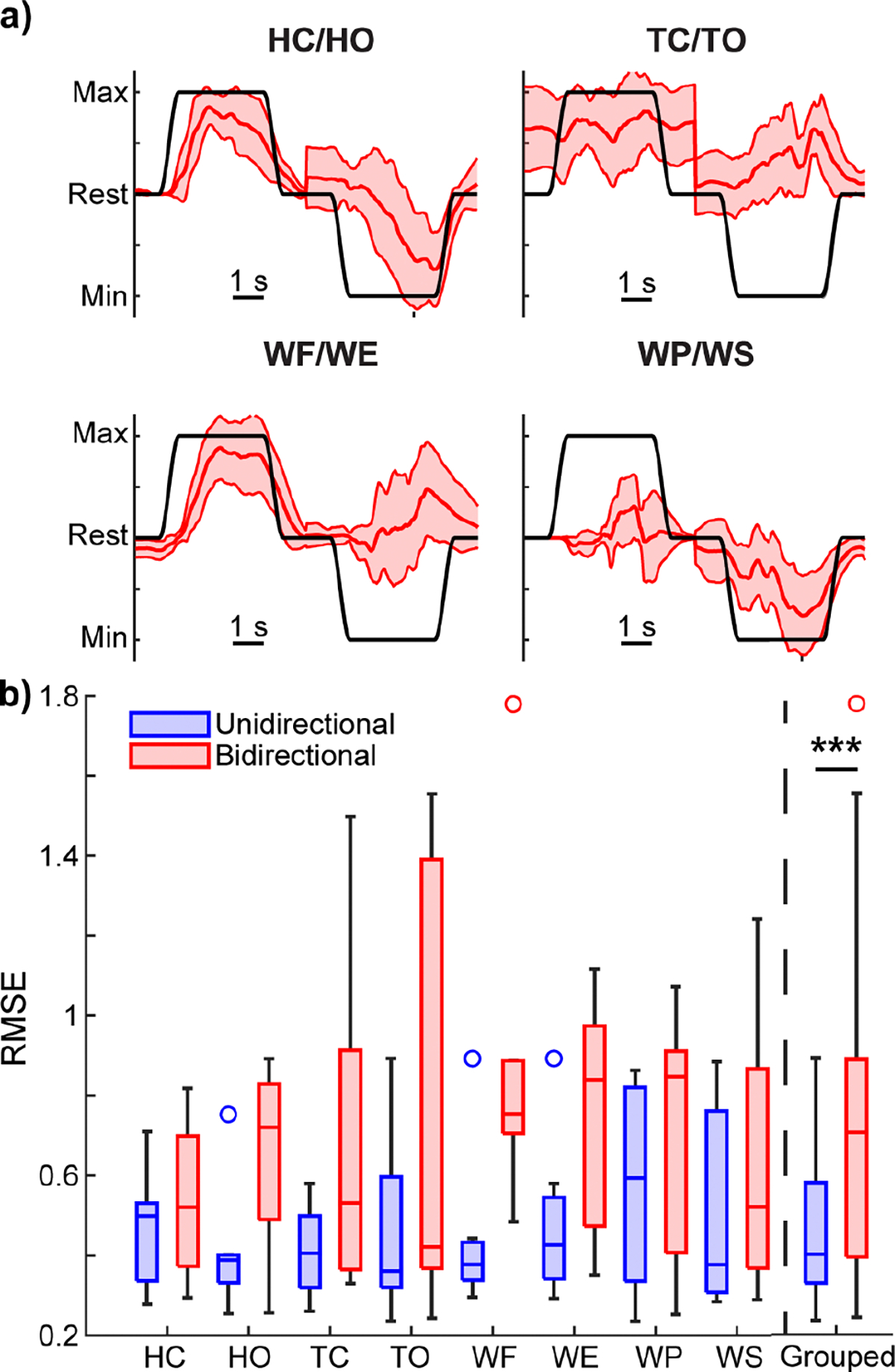
Bidirectional control of isolated hand and wrist motions. **a)** Kinematic predictions from one representative participant (the same participant from Fig 1a) for each of the movements. Red lines show the mean and standard deviation of the predicted kinematics across 10 trials of each movement from the testing dataset. Black lines show the ground-truth intended movement. The intended movement RMSEs for the example traces shown are as follows: HC = 0.44, HO = 0.72, TC = 0.40, TO = 1.20, WF = 0.48, WE = 1.01, WP = 0.85, WS = 0.53. Supplemental video S2 shows the mean traces for each movement. **b)** Boxplots of the average intended movement RMSE across participants for each movement under bidirectional control (red) or unidirectional control (blue, same data as shown in Fig. 3a). No significant differences were found at the individual movement level (*p* = 0.17, Kruskal-Wallis, N = 7 participants). However, when grouped across all movements, bidirectional movements performed significantly worse than unidirectional movements (*p* < 0.001 Wilcoxon signed-rank test, N = 56 (7 participants x 8 movements)). The overall median RMSE across all DOFs and participants was 0.40 for unidirectional control and 0.71 for bidirectional control. Boxplots show the median, interquartile range, and most extreme non-outlier values. Circles denote outliers.

With compelling evidence that the MKF could regress each movement in a unidirectional manner, we next explored whether the MKF could provide bidirectional control of each DOF (e.g., enabling both hand open and hand grasp). Performance was visually worse, with several overt errors in kinematic predictions (Fig. 4a, Supplementary Video S2). In several cases, the errors were directly opposite the intended movement. For example, when attempting to perform a tripod open, a tripod close was performed instead for one participant (Fig. 4a). Such an error would make it difficult, if not impossible, to release objects. Moreover, proportional position control is used for its ability to finely regulate force output, which is critical for fine motor tasks like manipulating a fragile object without breaking it. However, in this instance, errors directly opposing the user’s intent would substantially hinder fine force regulation and likely lead to broken objects. Grouped data across all motions support these visual observations; bidirectional movements performed significantly worse than unidirectional movements (Fig. 4b; *p* < 0.001, Wilcoxon signed-rank test).

### C. Two DOF Control Analysis

**Fig. 5.**
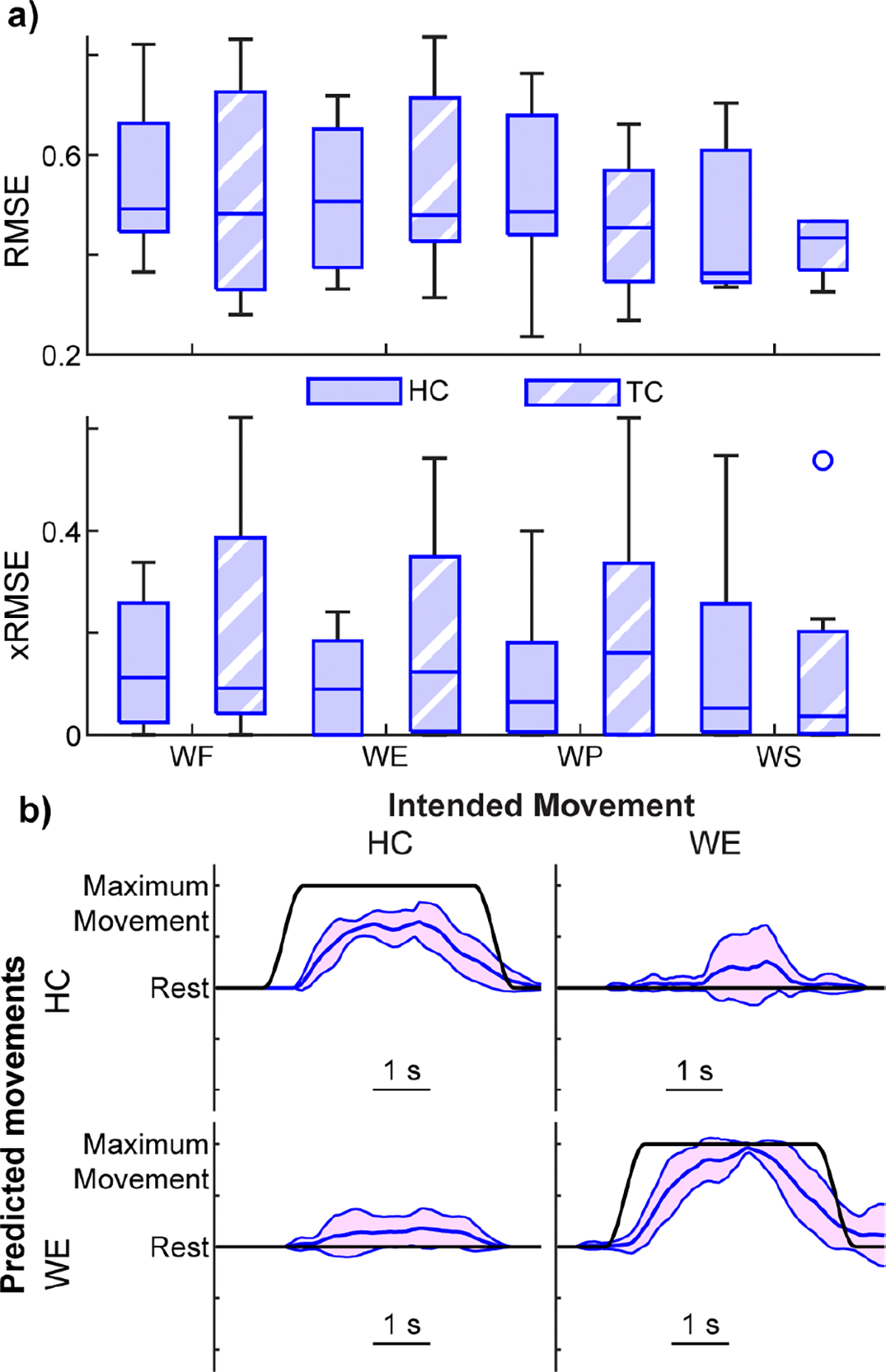
Unidirectional 2-DOF results. **a)** Boxplots of the median intended and unintended movement RMSE across participants for each wrist movement performed in conjunction with either HC (blue) or TC (blue striped). No significant differences were found for intended movement RMSE among the combinations (*p* = 0.77 Kruskal-Wallis). Similarly, no significant differences were found in unintended RMSE among the combinations (*p* = 0.98, Kruskal-Wallis). The median intended RMSE is 0.47, and the median unintended RMSE is 0.09. Data consist of one point per participant (N = 7). Boxplots show the median, interquartile range, and most extreme non-outlier values. Circles denote outliers. **b)** Kinematic predictions from a representative participant with an intended movement RMSE (0.51) near the median (0.47). The participant is the same participant shown in Fig. 3a. Blue lines show the mean and standard deviation of the predicted kinematics across trials of each movement from the testing dataset. Black lines show the ground-truth intended movement. Columns denote the intended movement; rows show the behavior of each DOF during those intended movements. The intended movement RMSEs for the example traces are HC = 0.55 and WE = 0.42. The unintended movement RMSEs for the example traces during each intended movement are HC = 0.10 and WE = 0.12. Supplemental video S2 shows the mean traces for each movement.

Commercially available myoelectric orthoses for hemiparetic participants only support a tripod pinch motion and do not restore any mobility to the wrist [11]. As such, we next explored how unidirectional grasping motions (i.e., HC or TC) could be used in conjunction with a functional wrist motion (i.e., WF, WE, WP, or WS). We found no difference among movement combinations for intended movement RMSE (Fig. 5a; *p* = 0.77, Kruskal-Wallis). Similarly, there was no significant difference among movement combinations for unintended movement RMSE (Fig. 5a; *p* = 0.98, Kruskal-Wallis). Across all movement combinations, the median intended movement RMSE was 0.47, and the median unintended movement RMSE was 0.09. These RMSE values are similar to those reported for amputees previously [53] but worse than those reported previously for healthy participants [72].

Visual observation of the kinematics also supports the use of 2-DOF control (Fig. 5b, Supplementary Video S2). For example, when using HC in combination with WE, intended movements are smooth and consistent, with only a minor variation in magnitude for HC. Similarly, unintended movements are minimal and relatively consistent, suggesting minimal impact on functional use.

### D. Three and Four DOF Analysis

**Fig. 6.**
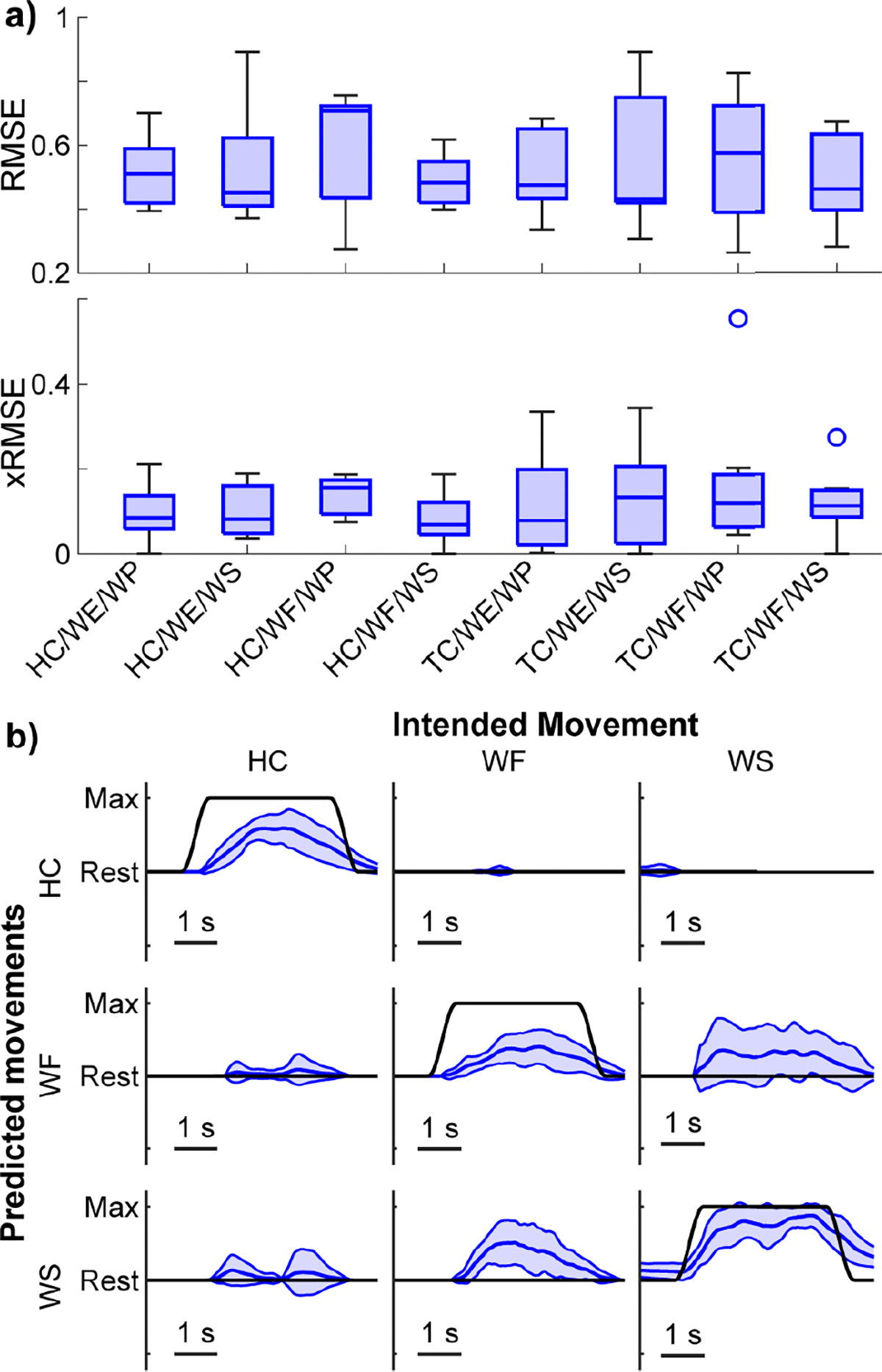
Unidirectional 3-DOF results. **a)** Boxplots of the median intended and unintended movement RMSE across participants for each 3-DOF combination. No significant differences were found for intended movement RMSE among the combinations (*p* = 0.94, Kruskal-Wallis). Similarly, no significant differences were found in unintended movement RMSE among the combinations (*p* = 0.81, Kruskal-Wallis). The median intended RMSE is 0.53, and the median unintended RMSE is 0.10. Data consist of one point per participant (N = 7). Boxplots show the median, interquartile range, and most extreme non-outlier values. Circles denote outliers. **b)** Kinematic predictions from a representative participant with an intended movement RMSE (0.55) near the median (0.53). The participant is the same participant shown in Fig. 3a. Blue lines show the mean and standard deviation of the predicted kinematics across trials of each movement from the testing dataset. Black lines show the ground-truth intended movement. columns denote the intended movement; rows show the behavior of each DOF during those intended movements. The intended movement RMSEs for the example traces are HC = 0.56, WF = 0.69, and WS = 0.37. The unintended movement RMSEs for the example traces during each intended movement are HC 0.02, WF = 0.18, and WS = 0.15. Supplemental video S2 shows the mean traces for each movement.

Given the feasibility of two controllable DOFs, we next explored the feasibility of three controllable DOFs. Here, we explore the combination of a grasping motion (i.e., HC or TC), with wrist flexion/extension (i.e., WF or WE), and with wrist rotation (i.e., WP or WS). Again, we found no significant difference between these combinations of movements in intended or unintended movement RMSE (Fig. 6a; *p*’s > 0.8, Kruskal-Wallis). Across all movement combinations, the median intended movement RMSE was 0.53, and the median unintended movement RMSE was 0.10. These RMSE values are worse than those reported previously for healthy and amputee populations [53], [72].

Visual observation of the median performing participant’s kinematics suggests 3-DOF control may be problematic for some hemiparetic participants (Fig. 6b, Supplementary Video S2). For example, when using HC in combination with WF and WP, intended movements are smooth and consistent, but there is considerable variation in magnitude for WF. More concerning, unintended movements are large and consistently coupled with another degree of freedom. Attempted WF leads to minimal WF and instead substantial HC and WS. Similarly, attempted WS leads to substantial WF. Such cross-talk would likely impact functional use.

**Fig. 7.**
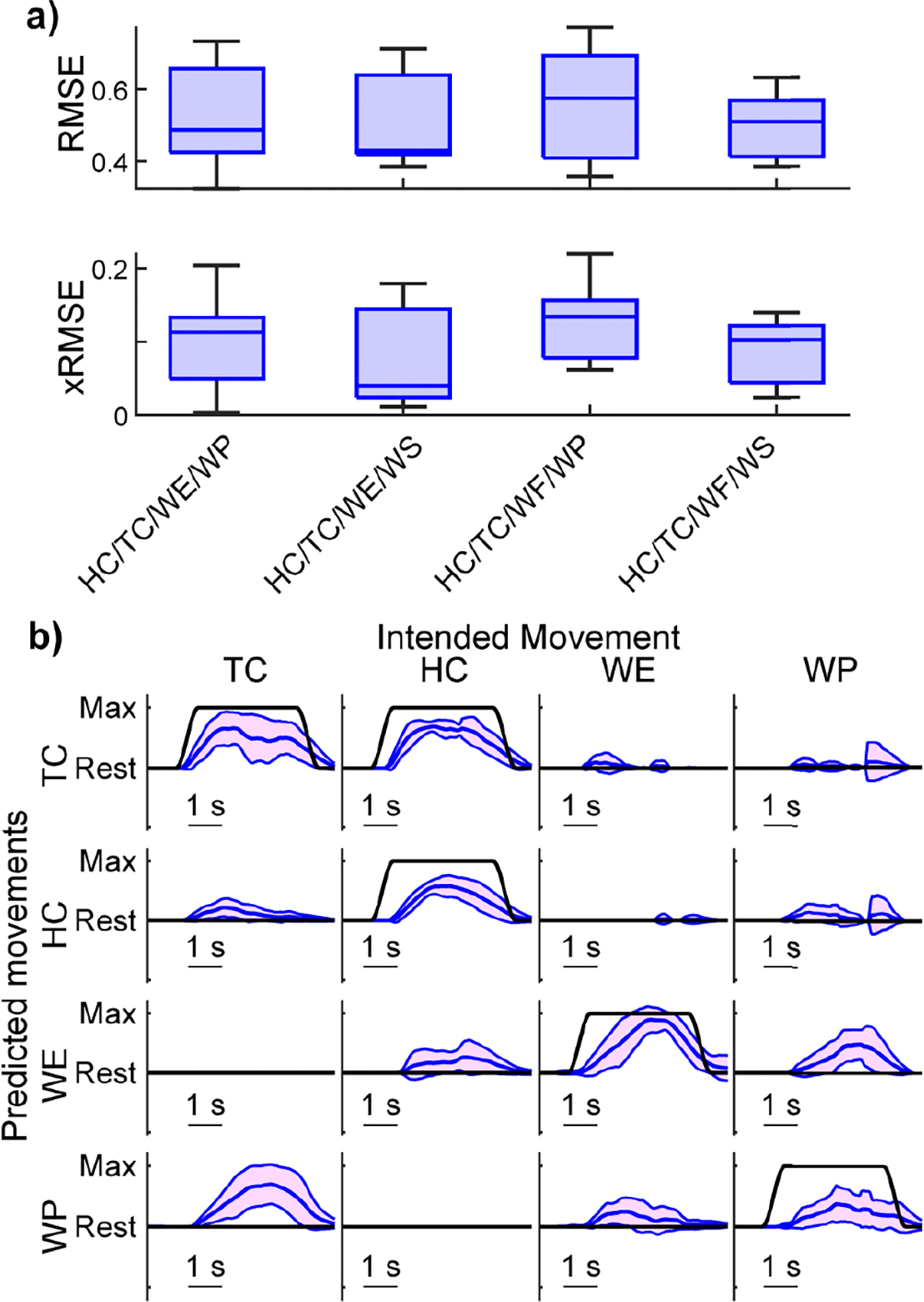
Unidirectional 4-DOF results. **a)** Boxplots of the median intended and unintended movement RMSE across participants for each 4-DOF combination. No significant differences were found for intended movement RMSE among the combinations (*p* = 0.90, Kruskal-Wallis). Similarly, no significant differences were found in unintended movement RMSE among the combinations (*p* = 0.43, Kruskal-Wallis). The median intended RMSE is 0.52, and the median unintended RMSE is 0.10. Data consist of one point per participant (N = 7). Boxplots show the median, interquartile range, and most extreme non-outlier values. **b)** Kinematic predictions from a representative participant with an intended movement RMSE (0.55) near the median (0.52). The participant is the same participant shown in Fig. 3a. Blue lines show the mean and standard deviation of the predicted kinematics across trials of each movement from the testing dataset. Black lines show the ground-truth intended movement. Columns denote the intended movement; rows show the behavior of each DOF during those intended movements. The intended movement RMSEs for the example traces are TC = 0.45, HC = 0.55, WE = 0.52, and WP = 0.77. The unintended movement RMSEs for the example traces during each intended movement are TC = 0.20, HC 0.07, WE = 0.07, and WP = 0.17.Supplemental video S2 shows the mean traces for each movement.

We also tested simultaneous and proportional position control of all four DOF combinations involving hand close, tripod close, wrist flexion/extension (i.e., WF or WE), and wrist rotation (i.e., WP or WS). This was the highest number of DOFs we investigated in this analysis. We found that each possible combination performed similarly in both intended and unintended movement RMSE (Fig. 7a, *p*’s > 0.4 Kruskal-Wallis). The median intended movement RMSE was 0.52, and the median unintended movement RMSE was 0.10.

Visual observation of the median performing participant’s kinematics suggests 4-DOF control may be problematic for some hemiparetic participants (Fig. 7b, Supplementary Video S2). Most intended movements are still clear and consistent, but the magnitude of intended WP is less than half of the desired amount. Similar to 3-DOF control, 4-DOF control has considerable unintended movement. For example, there is substantial and consistent unintended wrist pronation (WP) during tripod close (TC). There is also substantial wrist extension (WE) during wrist pronation (WP).

### E. Comparison of Multi-DOF Control

**Fig. 8.**
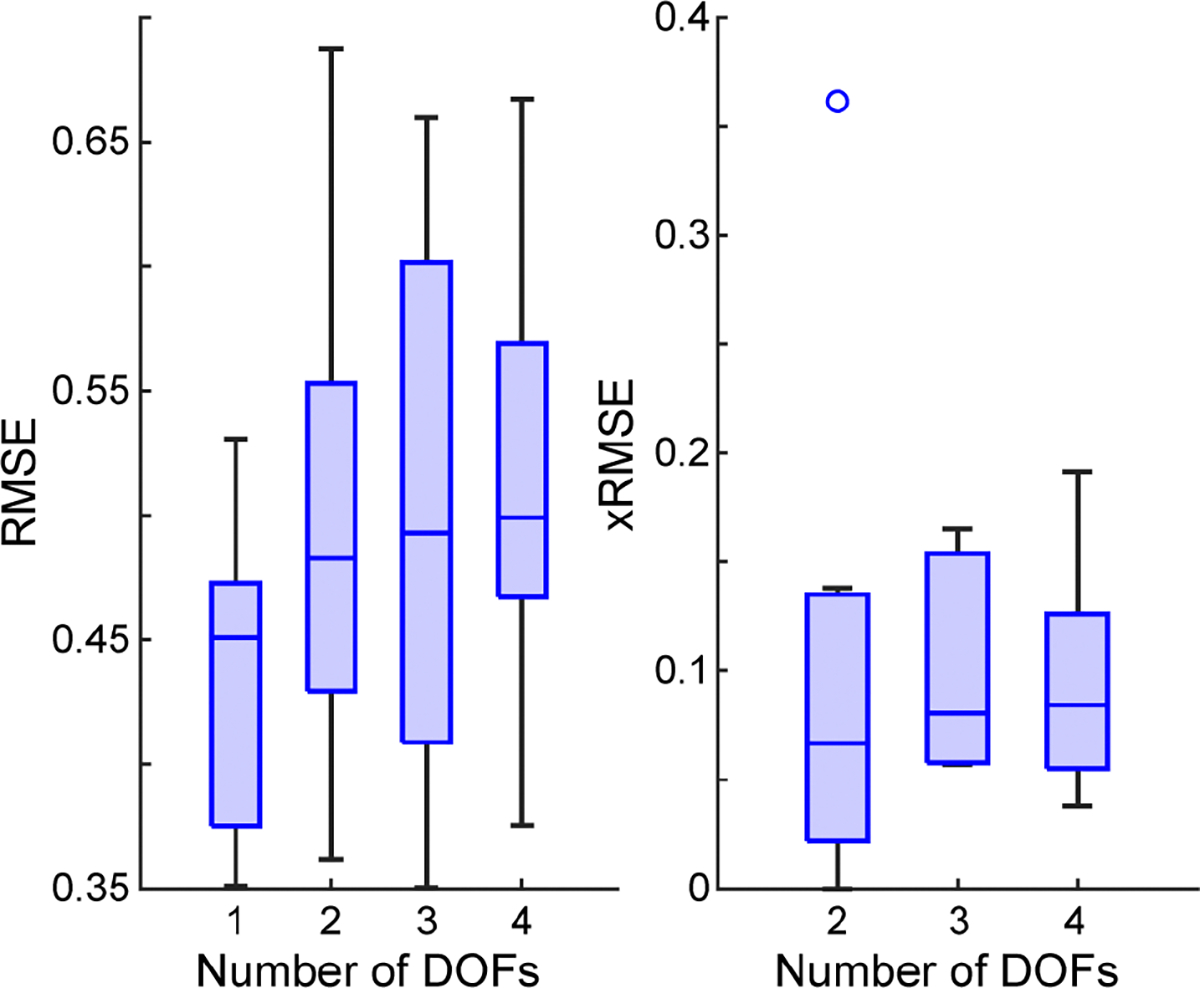
Performance as a function of DOFs. Boxplots of the median intended and unintended movement RMSE across participants for increasing numbers of controllable DOFs. No significant differences were found for intended movement RMSE (*p* = 0.38, Kruskal-Wallis). Similarly, no significant differences were found in unintended movement RMSE (*p* = 0.79, Kruskal-Wallis). Data consist of one point per participant (N = 7). Boxplots show the median, interquartile range, and most extreme non-outlier values. Circles denote outliers.

Consistent with prior work [72], both intended and unintended movement RMSE appear to increase as the number of controllable DOFs increases (Fig. 8). However, given the heterogeneity of hemiparesis and variability in participant performance, no statistical differences were found for intended and unintended RMSE (*p* = 0.38 and *p* = 0.79, respectively, Kruskal-Wallis).

Although higher DOF control was not significantly worse, visual observations of the kinematic outputs revealed that 3-DOF and 4-DOF control at the median level of performance are likely unusable (Supplementary Video S2). However, given the variability among participants and DOF combinations, we next sought to determine which participants, if any, might be able to achieve 3-DOF and 4-DOF control. To do this, we first identified the best-performing DOF combination from each participant, determined by the lowest intended and unintended RMSE. We then compared each participant’s best intended and unintended movement RMSE values against previously determined functional values from healthy participants [72]. The threshold for functional control was set as an intended RMSE of 0.45 and an unintended RMSE of 0.04 based on this prior work [72].

With this approach, we found that six of seven participants had at least one DOF combination that could achieve functional 2-DOF control (Table II). Across all seven participants, the best grasping motion was split; HC was optimal for four participants, and TC was optimal for three participants. Similarly, the best accompanying wrist motion varied; wrist rotation was optimal for five participants, and wrist flexion/extension was optimal for two participants.

Only four participants achieved functional control as we increased to 3-DOF control (Table III). For 3-DOF control, across all participants, HC was the best grasping motion for six of the seven participants. Wrist flexion and extension were split; WF was optimal for three participants, and WE was optimal for four participants. The optimal wrist rotation was supination for five participants. Among the four participants with functional control, HC was optimal for three participants, WE was optimal for three participants, and WS was optimal for three participants. All four participants with functional 3-DOF control had experienced an ischemic stroke.

**Fig. 9.**
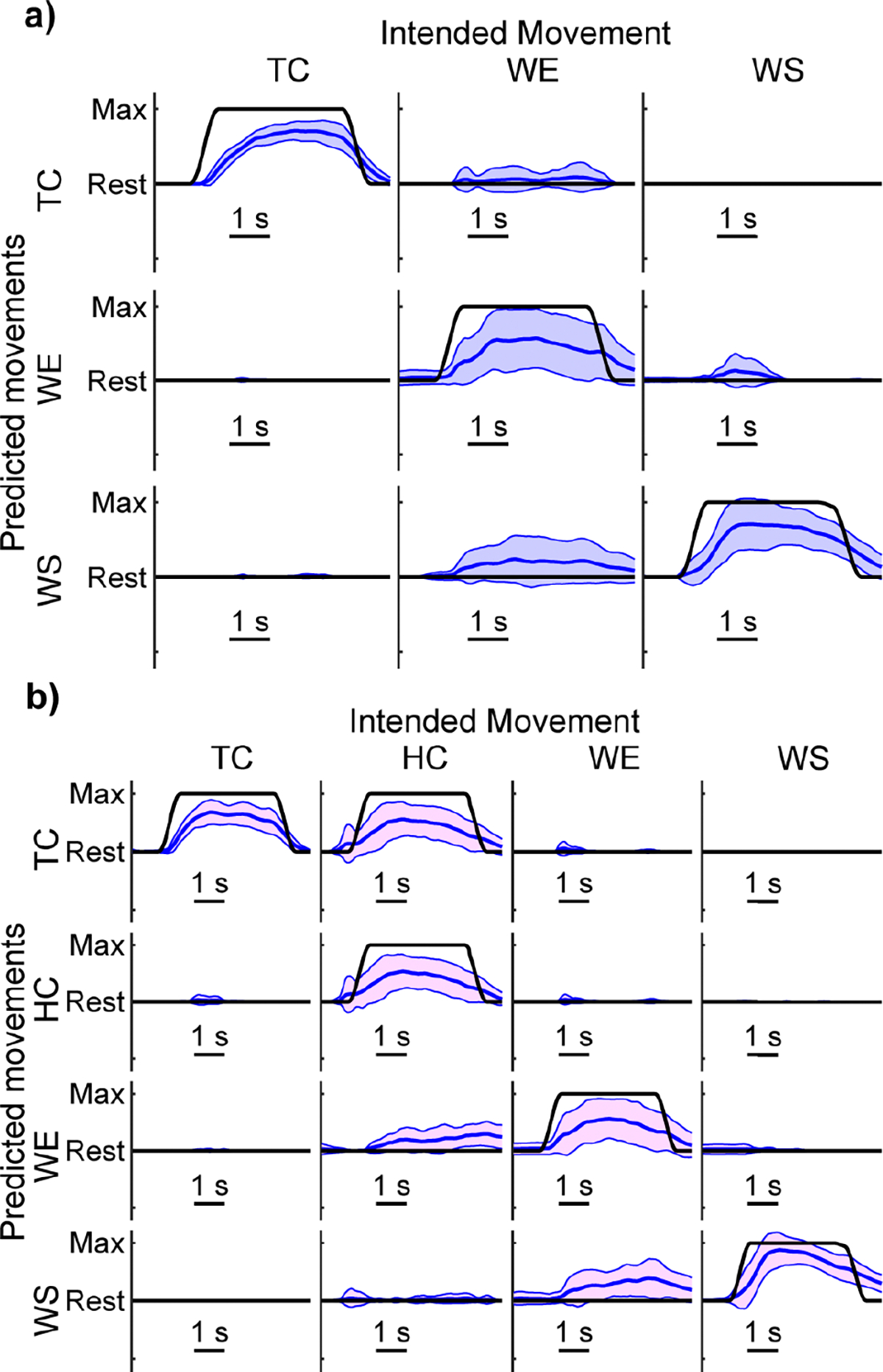
Kinematic predictions from the best performer. **a)** Kinematic predictions with an intended movement RMSE (0.43) and unintended movement RMSE (0.01) within the range determined to be functional. The intended movement RMSEs for the example traces are TC = 0.43, WE = 0.54, and WS = 0.41. The unintended movement RMSEs for the example traces during each intended movement are TC = 0.00, WE = 0.14, and WS = 0.01. **b)** Kinematic predictions with an intended movement RMSE (0.43) and unintended movement RMSE (0.01) within the range determined to be functional. The intended movement RMSEs for the example traces are TC = 0.42, HC = 0.56, WE = 0.51, and WS = 0.35. The unintended movement RMSEs for the example traces during each intended movement are TC = 0.00, HC = 0.13, WE = 0.11, and WS = 0.00. Blue lines show the mean and standard deviation of the predicted kinematics across trials of each movement from the testing dataset. Black lines show the ground-truth intended movement. Columns denote the intended movement; rows show the behavior of each DOF during those intended movements. Kinematic predictions are from the best performer, participant 5. Supplemental video S2 shows the mean traces for each movement.

Visual observation of the best-performing participant’s kinematics confirms that 3-DOF control is possible for some hemiparetic participants (Fig. 9a, Supplementary Video S2). For this participant, TC is remarkably consistent and has essentially no unintended movement associated with it. WE and WS are functional as well and have minimal unintended movement.

Only two participants were able to achieve functional 4-DOF control (Table IV). Again, the optimal DOF combinations varied across participants. WF was optimal for five participants, and WE was optimal for two participants. WS was optimal for four participants, and WP was optimal for three participants. The two participants who achieved functional control used the same DOF combination consisting of WE and WS.

Visual observation of the best-performing participant’s kinematics confirms that 4-DOF control is possible for some hemiparetic participants (Fig. 9b, Supplementary Video S2). TC is remarkably consistent and reliably differentiable from HC, even though TC exists as a subset of HC. HC, WE, and WS are also functional and have minimal unintended movement.

In summary, 86% of participants achieved functional 2-DOF control, 57% achieved functional 3-DOF control, and 29% achieved functional 4-DOF control. This represents a 29% drop in participants for each additional controllable DOF. Only participants 4 and 5 achieved 4-DOF control; both suffered an ischemic stroke and an MAS score of 2.

## IV. Discussion

This work represents the first investigation of simultaneous and proportional myoelectric position control of multiple hand DOFs for hemiparetic patients. We build on prior works that have shown multi-class classification [7], [38], [39] and single-DOF regression [59] to explore multi-DOF regression in hemiparetic patients. We demonstrate that single-DOF control is effective across multiple different hand and wrist motions, and that performance is optimal when DOFs are limited to a single unidirectional motion instead of two opposing bidirectional motions. Furthermore, we demonstrate that functional 2-DOF is possible for the majority of hemiparetic patients. 3-DOF and 4-DOF control are also possible but appear to be only achievable by smaller subsets of patients.

The ability to simultaneously control grasping and wrist motions could have an immediate impact on the dexterity of hemiparetic patients using assistive myoelectric exoskeletons. With new reimbursement pathways [73], it is anticipated that assistive myoelectric exoskeletons will become more common among the large populations of patients with hemiparesis. As such, this work provides initial proof of concept that these devices could restore even more dexterity and can serve as motivation to develop active wrist modules and higher-density EMG arrays into these powered upper-limb orthoses.

Future work should validate these initial findings in a larger cohort of hemiparetic stroke patients. We found no significant difference in performance as the number of DOFs increased and no significant differences among the many different DOF combinations, likely due to the heterogeneity of hemiparesis and the limited sample size. However, lack of statistical significance does not indicate equivalence [74]. Larger cohorts of patients may be able to find broader trends across patients, but given the heterogeneity of hemiparesis, control strategies will likely still need to be tailored to each patient, as is common with myoelectric control and orthotics more broadly.

Future work should also explore real-world multi-DOF regression control of an assistive exoskeleton where the participants are actively in the loop. Prior work has suggested that offline performance does not necessarily indicate online performance [75], [76]. However, several studies have also shown that offline analyses, like the present study, are useful and can indicate online performance [38], [53], [63], [77]. The promising offline results reported here constitute an important step towards functional gains with a more dexterous, assistive exoskeleton.

Long-term learning and improvement also warrant further investigation. Only two of the seven participants were able to achieve 4-DOF control in this study; it is possible that the other five patients could achieve 4-DOF control with additional training. There is an element of learning involved in online myoelectric control [63], [76], and the fact that hemiparetic EMG can be improved [14], [15] implies that control could be improved over time. Additional training also leads to more training data, which has been shown to improve multi-DOF regression [63], [68]. Given the custom nature of orthotics, it is possible that EMG recordings could be consistent across days, as has been shown with the high-density sleeves and armbands such as the one used in this study [38], [65]. Long-term use would allow for continued human learning as well as continued machine learning, where more complex actions are incorporated into the algorithm over time [78].

Although the sample size is limited, three interesting discussion points arise from the patient demographics. First, we found no significant difference among unidirectional motions. One might have expected to see worse performance for extension motions (e.g., HO, TO), given that extension is diminished in hemiparesis [79]. Instead, the results presented here corroborate prior work showing similar levels of myoelectric control for both flexion and extension movements in hemiparetic stroke patients [59]. Nevertheless, we did see worse performance with bidirectional control, which is likely attributed to diminished extensors and impaired coordination and recruitment of agonist muscles [80].

Second, we observed that only some patients were able to achieve functional 3-DOF and 4-DOF control. An important question is whether or not this subset of patients differed from the other patients in any way. Although the sample size is limited, the main difference between those with functional 3-DOF control and those who did not was that those who had functional control suffered an ischemic stroke, whereas those who did not suffered either a hemorrhagic stroke or TBI. However, the other characteristics, like the time since injury and MAS score, were variable. Similarly, we found no visually apparent differences in EMG activity among these patients.

Third, on that point, we found no differences between the patients with MAS scores of 1 and the patients with MAS scores of 2. Prior studies have shown that more impaired stroke participants, or those with less observable movement, had lower myoelectric classification performance [38], [81]. In contrast, our results corroborate prior work that showed no correlation between MAS scores and 1-DOF regression performance [59]. The present study focused on patients with MAS scores of 2 or lower, as this is the patient population currently using assistive myoelectric exoskeletons. However, prior work has shown that patients with MAS scores of 3 are able to achieve at least 1-DOF control at similar levels to other stroke participants and healthy controls [59]. Future research should explore multi-DOF control in this patient population, as these individuals stand the most to gain from more dexterous assistive technologies.

In this work, we defined functional control based on the performance of healthy individuals reported previously [72]. Although applying a fixed threshold could be viewed as somewhat arbitrary, visual observations support the criteria selected. There are stark differences between the median performance and best performance for 3-DOF control (Fig. 4b and Fig. 7a) and for 4-DOF control (Fig. 5b and Fig. 7b). The predominant difference arises from a relatively small change in unintended movement RMSE (e.g., from ~0.10 to ~0.01). Unintended movement RMSE is inherently smaller than intended movement RMSE since it is averaged across DOFs and purposefully reduced by algorithmic design [53]. Nevertheless, the reduction in unintended movement RMSE is still an order of magnitude difference that determines functionality.

In this study, we used RMSE as an outcome measure. RMSE has been used previously in the development of myoelectric control for prostheses [82], [83], [84] and exoskeletons [85], [86]. Additionally, for offline studies, RMSE and other offline performance metrics have been shown to be representative of online control [63], [77]. Nevertheless, it is important to link RMSE values to functional improvements. In one study, a drop of RMSE from about 0.16 to 0.14 was correlated with a decrease in the average number of drops in force from 10 to seven [87]. Another study reported RMSE values around 12% of maximum voluntary contraction yielded around 15 transfers in the box and blocks task and 10 s per clothespin transfer in the clothespin relocation task [88]. Although there is no direct link to a specific RMSE value and the functional outcomes an individual achieves, promising RMSE values can provide a good initial way to assess algorithms before incorporating patients into the loop [82]. Minimizing the time requested of patient time is particularly important when developing assistive technology for stroke patients [62].

It is also important to compare this work to other potential control algorithms. Classification, or gesture recognition, has been used [7], [38], [39], however, comparisons between discrete approaches, including classification and continuous approaches like proportional control, are limited.

No studies to the knowledge of the authors investigate proportional control of the hand and wrist with stroke patients. However, proportional control algorithms are common in myoelectric prostheses. Some proportional control algorithms that have been used in the field of myoelectric prostheses include convolutional neural networks, multilayer perceptrons, and long short-term memory networks [83]. Dantas et al. investigated these and reported RMSE values between 0.2 and 0.3 [83]. From the best performing movement combinations, we report intended RMSE values between 0.3 and 0.45 and unintended RMSE values less than 0.04. Averaging these values together suggests that our approach offers comparable performance. Future work should investigate these other proportional control algorithms for exoskeleton control.

Another approach to control multiple DOFs from EMG is to use neuromuscular modeling [29], [30]. This has been demonstrated with healthy [29], [34] and amputee populations [30] with RMSEs between 0.2 and 0.3. Here, the reported RMSE values suggest that our approach offers comparable performance for the best-performing movement combinations. Importantly, the approach used here did not rely on any prior information about the neuromuscular dynamics of the individual. Neuromuscular modeling would likely need to be adapted to account for the weak, spastic, and uncoordinated muscle activity found in the paretic limb of stroke patients [17], [19], [21], [22].

Given that large unintended movements became more common across patients as the number of DOFs increased, one might conclude that simultaneous regression is not ideal for paretic EMG. However, the classification accuracy also decreases as the number of gestures/motions increases. True simultaneous control of 4-DOFs gives rise to a near-infinite number of classes. Even if each of the four DOFs is discretized into three positions, one would still need 81 possible classes. It is likely that the same population capable of achieving adequate classification accuracy with a large number of classes would also be candidates for 4-DOF regression control, and prior work has shown that myoelectric regression outperforms classification [44].

This work and prior works have largely applied existing algorithms from the fields of prosthetics or human-computer interaction to hemiparetic EMG. Ultimately, new models of control may be needed for this unique patient population and application. Combining classification and regression might allow better control for assistive devices than either approach individually. This has been done previously by combining a multilayer perceptron classifier with a Kalman filter-based decoder for prosthetic control [89]. This work found that a linear combination of the two algorithms took the benefits of both algorithms and reduced the negatives, effectively providing regression with minimal jitter and minimal unintended movements [89]. Such an approach may be suitable for hemiparetic patients, where fatigue, spasticity, and co-contractions can lead to more unintended movements relative to healthy and amputee populations.

## V. Conclusion

Simultaneous and proportional control of multiple DOFs is possible for myoelectric prostheses and human-computer interfaces. This work demonstrates that a common regression algorithm, a Kalman filter, can readily be applied to paretic EMG to provide hemiparetic patients with simultaneous and proportional multi-DOF control. Such control could enable more dexterous assistive exoskeletons that improve the quality of life for individuals with hemiparesis.

## Supplementary Material

supp2-3599062

supp1-3599062

This article has supplementary downloadable material available at https://doi.org/10.1109/TNSRE.2025.3599062, provided by the authors.

## Figures and Tables

**TABLE I:** Participant Demographics

Participant	Age	Sex	MAS	Injury	Years since injury
**1**	32	M	2	TBI	10.8
**2**	54	M	2	Ischemic Stroke	6.3
**3**	44	F	1	Hemorrhagic Stroke	3.8
**4**	47	M	2	Ischemic Stroke	1.7
**5**	66	M	2	Ischemic Stroke	1.2
**6**	47	M	1	Ischemic Stroke	1.4
**7**	30	M	2	TBI	6.6
**Average ± STD**	45.7 ± 12.4	86% M	1.7 ± 0.5		4.5 ± 3.6

**TABLE II:** Best Two DOF Combinations

Participant	Combo	RMSE	xRMSE
**1**	HC/WS	0.36	0.02
**2**	HC/WS	0.33	0
**3**	HC/WP	0.24	0
**4**	TC/WS	0.33	0
**5**	TC/WP	0.34	0
**6**	TC/WE	0.31	0
**7**	HC/WF	0.48	0.11

**TABLE III:** Best Three DOF Combinations

Participant	Combo	RMSE	xRMSE
**1**	HC/WF/WS	0.55	0.13
**2**	HC/WF/WS	0.40	0
**3**	HC/WE/WP	0.39	0.06
**4**	HC/WE/WP	0.42	0.0006
**5**	TC/WE/WS	0.43	0.01
**6**	HC/WE/WS	0.37	0.04
**7**	HC/WF/WS	0.48	0.09

**TABLE IV:** Best Four DOF Combinations

Participant	Combo	RMSE	xRMSE
**1**	HC/TC/WE/WP	0.55	0.12
**2**	HC/TC/WE/WP	0.32	0.11
**3**	HC/TC/WF/WP	0.35	0.13
**4**	HC/TC/WE/WS	0.42	0.04
**5**	HC/TC/WE/WS	0.43	0.01
**6**	HC/TC/WE/WS	0.39	0.05
**7**	HC/TC/WF/WS	0.63	0.14
