# Supplementary material for: Simultaneous and Proportional Myoelectric Control of Multiple Degrees of Freedom in Individuals With Chronic Hemiparesis
Source: IEEE Trans Neural Syst Rehabil Eng. Author manuscript; Available in PMC 2025 Sep 12. (PMC12425881; doi:10.1109/TNSRE.2025.3599062)
Supplement: supp1-3599062 [file NIHMS2106910-supplement-supp1-3599062.docx]

SUPPLEMENTARY TABLE SI

Kruskal-Wallis Test Results

| Comparison | *p* value |
| --- | --- |
| 1 DOF movement | 0.98 |
| 1 DOF participant | 0.45 |
| Unidirectional vs Bidirectional | 0.17 |
| 2DOF RMSE | 0.77 |
| 2DOF xRMSE | 0.98 |
| 3DOF RMSE | 0.94 |
| 3DOF xRMSE | 0.81 |
| 4DOF RMSE | 0.9 |
| 4DOF xRMSE | 0.43 |
| RMSE vs DOF | 0.38 |
| xRMSE vs DOF | 0.79 |

Supplementary Video S2

Video Representations of the average traces shown in Figs 3, 4, 5, 6, 7, and 9 using a MuJoCo hand (Google DeepMind, London, United Kingdom). The traces appear in the order of the figures, with the trace being represented in the MuJoCo hand on the left and the kinematic trace represented in plots on the right, where black shows the intended kinematics and red shows the predicted kinematics. First clip: unidirectional 1-DOF control showing the average kinematic trace in Fig. 3 in the order of hand close (HC), hand open (HO), tripod close (TC) tripod open (TO), wrist flexion (WF), wrist extension (WE), wrist pronation (WP) and wrist supination). Second clip: bidirectional 1-DOF control showing average traces from figure 4, HC and HO, TC and TO, WF and WE, WP and WS. Third Clip: Two-DOF control from Fig. 5. HC and WE. Fourth clip: Three DOF control from Fig. 6, HC, WF, and WS. Fifth clip: Four DOF control from Fig. 7, TC, HC, WF and WP. Sixth clip: Good 3-DOF control from Figu 9a. TC, WE, and WS. Seventh clip: Good 4-DOF control from Figure 9b, TC, HC, WE, and WS.
